# Symptom Preoccupation in Atrial Fibrillation and its Association With Quality of Life: A Cross-Sectional Study

**DOI:** 10.1016/j.jacadv.2025.102058

**Published:** 2025-08-08

**Authors:** Björn Erik Liliequist, Brjánn Ljótsson, Eva Ólafsdóttir, Frieder Braunschweig, Josefin Särnholm

**Affiliations:** a Division of Psychology, Department of Clinical Neuroscience, Karolinska Institutet, Stockholm, Sweden; b Department of Medicine, Cardiology Unit, Karolinska Institutet Karolinska University Hospital, Stockholm Sweden, Karolinska University Hospital Solna, Stockholm, Sweden; c Department of Cardiology, Karolinska University Hospital, Stockholm Sweden, Karolinska University Hospital Solna, Stockholm, Sweden; d Institution of Medicine, Cardiology Unit, Karolinska Institute, H7 Medicine, Huddinge, Stockholm, Sweden; e Department of Cardiology, Heart and Vascular Center, Karolinska University Hospital, Karolinska University Hospital Huddinge, H7 Medicine, Stockholm, Sweden; f Center for Behavioral Cardiovascular Health, Columbia University Irving Medical Center, New York, New York, USA

**Keywords:** atrial fibrillation, arrhythmia, quality of life, symptom preoccupation

## Abstract

**Background:**

Atrial fibrillation (AF) often manifests with distressing symptoms, impaired quality of life (QoL), and increased health care consumption. Rhythm and rate control treatments may have limited impact on QoL. Symptom preoccupation, that is, fear of cardiac-related symptoms and avoidance behavior, may contribute to disability in AF but remains understudied.

**Objectives:**

The objective of the study was to investigate the prevalence of symptom preoccupation in AF and its association with impaired QoL, general disability, symptom severity, and health care visits, in a general AF sample.

**Methods:**

This cross-sectional study recruited 409 Swedish AF patients who completed an online survey on demographics, medical history, and measures of AF-specific QoL (AF effects on QoL; AFEQT), general disability, AF health care use, and symptom preoccupation (Cardiac Anxiety Questionnaire) and other psychological variables, including depression and general anxiety.

**Results:**

Symptom preoccupation was present in 37% of respondents (59% females and 23% males) and showed the strongest association with impaired QoL (regression coefficient [β]: −0.51; 95% CI: −0.61 to −0.41; *P* < 0.001), health care use (incidence rate ratio: 1.67; 95% CI: 1.28-2.19; *P* < 0.001), and symptom severity (β: 0.39; 95% CI: 0.27-0.50; *P* < 0.001). Depression was most strongly associated with general disability (β: 0.45; 95% CI: 0.32-0.58; *P* < 0.001), whereas symptom preoccupation also showed a significant but weaker association (β: 0.31; 95% CI: 0.19-0.43; *P* < 0.001) with general disability.

**Conclusions:**

Symptom preoccupation is common in AF patients. Behavioral interventions that specifically target symptom preoccupation could potentially increase QoL, reduce health care use, and improve symptom experience in AF.

Atrial fibrillation (AF) is a prevalent arrhythmia affecting 3% of the adult population.^1^ AF often presents with disabling symptoms, such as palpitations, fatigue, dyspnea, and dizziness,^2^^,^^3^ leading to impaired quality of life (QoL),^4^ particularly among female patients,^5^ and increased health care consumption.^4^ Importantly, many patients with AF experience impaired QoL and persistent symptoms, indicating limitations of current treatment strategies.^6^^,^^7^ Consequently, identifying factors beyond arrhythmia burden that influence disability in AF could help develop more comprehensive and effective approaches to AF care.

Depression and anxiety are known to contribute to low QoL,^8^ greater self-rated symptom severity,^9^ and increased health care seeking^4^ in AF. Anxiety sensitivity^10^ has been linked to more severe self-rated AF symptoms, whereas perceived stress^11^ is often reported as a trigger for the onset of AF symptoms. Consequently, there have been calls for approaches to AF care that include psychosocial interventions AF care.^12^^,^^13^ However, disease-specific factors such as symptom preoccupation^10^^,^^14^ may also play a significant role in AF disability. Symptom preoccupation encompasses fear of cardiac-related symptoms, AF-related avoidance, and control behavior, for example, excessive pulse checking and avoidance of social and physical activities in fear of experiencing or eliciting AF symptoms, as well as hypervigilance toward the heart.^14^ These behavioral factors can negatively affect both symptom experience and daily functioning in AF. For example, hypervigilance toward AF symptoms can amplify the perception of cardiac-related symptoms, which may overlap with anxiety symptoms like palpitations and shortness of breath.^15^ Furthermore, avoidance of daily activities is likely to decrease QoL. Evidence suggests symptom preoccupation is associated with lower QoL in AF,^10^ and AF-specific cognitive behavioral therapy (AF-CBT) protocol, targeting symptom preoccupation in paroxysmal AF, has shown significant improvements in AF-specific QoL and reductions in AF-related health care consumption.^16^ Preliminary evidence also suggests that reduced symptom preoccupation mediated the improvement in AF-specific QoL following AF-CBT.^17^

Despite these findings, research on the prevalence of symptom preoccupation and its interplay with general psychological factors like depression and anxiety in a broader AF population is limited. The purpose of this cross-sectional study of a general AF sample was therefore to: 1) investigate the prevalence of symptom preoccupation in the total sample and among women and men; and 2) to further investigate the role of symptom preoccupation and general psychological factors in AF-disability. We investigated the associations between symptom preoccupation compared to depression, general anxiety, fear of bodily symptoms, anxiety sensitivity, and perceived stress, with: 1) AF-specific QoL; 2) general disability; 3) self-rated AF symptom severity; and 4) AF-specific health care consumption. We also wanted to investigate if the different facets of symptom preoccupation, that is, cardiac-related fear, hypervigilance toward cardiac-related symptoms, and AF-related avoidance behavior, were associated with impaired AF-specific QoL.

## Methods

### Study design

This cross-sectional study was conducted at the Karolinska University Hospital in Stockholm, Sweden. Participants with an AF diagnosis were recruited nationally via self-referral from clinics, newspaper, and social media advertisement. Data were collected at a single time point in March 2020 through an online survey. The study was approved by the Swedish ethical review authority (number: 2019-03365) and was conducted in accordance with the Declaration of Helsinki. All participants provided informed consent.

### Respondents

Respondents aged 18 years or older, with an established diagnosis of paroxysmal, intermittent, or permanent AF, and able to read and write in Swedish were eligible for inclusion in the study.

### Recruitment and procedure

Study information was distributed to waiting rooms at 27 cardiology clinics across Sweden through an advertisement in a free of charge newspaper reaching all households in the greater Stockholm area, and through social media. The study information read, “Do you have atrial fibrillation? Would you like to participate in a survey study on what it's like to live with atrial fibrillation?”. Respondents registered online on the study webpage, completed a digital informed consent, and completed self-rated assessments via a secure web-based assessment tool. Respondents were offered compensation with a voucher with the value of 200 SEK (approximately $20).

## Measurements

### Diagnosis and classification of atrial fibrillation

Respondents were asked to provide the date of their AF diagnosis, whether their AF was currently managed in primary or tertiary care, and to confirm the type of AF diagnosis (paroxysmal, persistent, permanent, or unknown) they were diagnosed with. Self-report of somatic diagnosis has demonstrated accuracy in studies of other chronic diseases.^18^ See the Supplemental Appendix for more details.

### Primary independent variable

The Cardiac Anxiety Questionnaire (CAQ)^19^ was used to measure symptom preoccupation in AF. The CAQ consists of 18 items divided in 3 subscales measuring different dimensions that correspond to the concept of symptom preoccupation; cardiac-related fear (eg, “I worry that I may have a heart attack”), attention/hypervigilance to cardiac-related symptoms (eg,“I pay attention to my heart beat”) and cardiac-related avoidance behavior (eg, “I avoid physical exertion”). Items are rated on a 5-point scale from 0 (“never”) to 4 (“always”). The total score ranges from 0 to 72, with a greater score representing more symptom preoccupation. We defined symptom preoccupation as a score of ≥26, based on the criteria suggested by Van der Beek et al, which indicates clinically significant levels of cardiac anxiety analogous to symptom preoccupation.^20^

### Secondary independent variables

The Patient Health Questionnaire (PHQ-9)^21^ is a 9-item measure of depression and severity of depressive symptoms. Items are rated on a 4-point scale from “not at all” to “nearly every day”. Scoring ranges from 0 to 27 with a higher score indicating more severe depressive symptoms, with cutoff for clinically significant depressive symptoms at ≥10 points.^22^ The General Anxiety Disorder-7^23^ is a 7-item measure of generalized anxiety with a scoring ranging from 0 to 21 with higher scoring indicating higher levels of generalized anxiety. Cutoff for clinically significant levels of general anxiety have been set at ≥8 points.^22^ The Body Sensations Questionnaire^15^ measures fear of bodily sensations and consists of 17 items with a scoring ranging from 0 to 72 with a greater score indicating more fear of bodily symptoms. The Anxiety Sensitivity Questionnaire^24^ consists of 16 items measuring anxiety sensitivity with a total score of 0 to 64, with a higher score indicating higher levels of anxiety sensitivity. The Perceived stress scale^25^ consists of 4 items measuring stress reactivity with a total score ranging from 0 to 16, with higher numbers indicating higher levels of perceived stress.

### Dependent variables

#### Quality of life

The AF effects on QoL (AFEQT)^26^ consists of 16 items measuring AF-specific QoL in the following 4 dimensions: 1) symptom severity; 2) physical and social impairment; 3) AF-related worrying; and 4) concern and satisfaction with current medical treatment regimen. The total score ranges between 0 and 100 with lower scores indicating lower AF-specific QoL. Spertus et al defined AF severity as follows: mild 71.3 (±19.2), moderate 57.9 (±19.0), and severe 42.0 (±21.2).^26^

#### General disability

General disability was measured with the 12-item version of the World Health Organization Disability Assessment Schedule (WHODAS 2.0).^27^ WHODAS 2.0 measures disability in the following 6 domains: 1) cognition; 2) mobility; 3) self-care; 4) getting along (interaction with other people); 5) life activities; and 6) participation in community activities. The summary score ranges from 0 to 100 with a higher score indicating more disability.

#### Symptom severity and AF-specific health care consumption

Severity of the AF-related symptom was measured with the University of Toronto Atrial Fibrillation Severity C-Scale (AFSS)^28^ consisting of 7 items measuring severity of AF-related symptoms (eg, “palpitations: how often have you been bothered by this symptom during the last 4 weeks?”). The total scoring ranges from 0 to 35 with a higher score indicating more severe symptoms. AF-related health care visits were measured using 3 items from the AFSS, measuring the number of emergency room visits, visits to cardiologist in specialized health care, and hospital admissions due to AF over the last 3 months.

### Covariates

The following sociodemographic variables and medical covariates were collected as self-report, a method that has been shown to be acceptable:^18^ age, sex, highest achieved education (prehigh school, high school, posthigh school, university, or doctoral degree), years since AF diagnosis, type of AF, self-reported frequency of AF episodes, angina pectoris, heart failure, coronary heart disease/previous myocardial infarction, hypertension, chronic obstructive pulmonary disease, sleep apnea, previous stroke, body mass index, diabetes, thyroidism, disturbed sleep, presence of functional somatic disorder (eg, fibromyalgia or irritable bowel syndrome), total number of medications, and presence of any other medical condition.

### Statistical analysis

Sample characteristics were summarized as mean and SD for continuous variables and numbers and percentages for categorical variables. The associations between the primary (symptom preoccupation) and secondary (depression, general anxiety, fear of bodily symptoms, anxiety sensitivity, and perceived stress) independent variables and the outcomes were investigated in a series of regression analyses, controlling for all covariates. First, 3 linear multiple regression analyses were performed to assess the influence of the independent variables on the outcome of AF-specific QoL, general disability, and AF-symptom severity, respectively. Then, we used Poisson regression to assess the influence of the independent variables on AF-specific health care use. Results for the Poisson are reported as both regression coefficients and incidence rate ratios (IRRs) with corresponding *P* values and 95% CI. Finally, a linear regression analysis investigated the independent influence of the 3 CAQ subscales cardiac-related fear (fear), attention/hypervigilance to cardiac-related symptoms (hypervigilance), and cardiac-related avoidance behavior (avoidance) on AF-specific QoL, controlling for the secondary independent variables and covariates as detailed above. Model assumptions were assessed using residual plots, Q–Q plots, and Cameron and Trivedi's decomposition of the information matrix test. Some skewness was observed in the AF symptoms severity model, and robust SEs were applied in all regression analyses to ensure valid inference. Multicollinearity was ruled out (maximum variance inflation factor < 5). For the Poisson regression, the model fit and dispersion were evaluated using Pearson goodness-of-fit tests and by comparing with a negative binomial model, which supported the use of Poisson. All continuous variables were standardized before analysis. This allowed us to compare the effects across variables that may be measured on different scales or in different units.

The results of the linear multiple regression analyses were reported as regression coefficients (β) with 95% CI and *P* values to explore how much the outcome changed in units of SDs for a 1-SD change in the standardized independent variable, and for the presence of a binary covariate. F-statistics, with corresponding *P* values, were calculated to assess the overall model fit, and we used the determination coefficient (R^2^ change), which represents the proportion of variance explained by the primary and secondary independent variables and the covariates.

In the Poisson regression model for AF-specific health care consumption, the results were reported as IRRs with *P* values and corresponding 95% CIs. The IRR presents the impact measured in SDs caused by a change of 1-SD in the independent variable. Wald's Chi-squared test and its corresponding *P* value were used to assess the overall fit of the model, and the pseudo-R2 coefficient was used to assess the proportion of variance explained by the primary and secondary independent variables and the covariates. Robust SEs were used in all regression analyses to account for heteroskedasticity and improve the reliability of the estimated coefficients. These are reported in the tables but omitted from the text for clarity. For transparency, βs with corresponding 95% CIs are also reported in the Poisson regression tables.

## Results

### Participant enrollment and recruitment sources

A total of 443 individuals registered for participation in the study, 32 applicants were excluded due to not completing the questionnaires, 2 applicants declined participation, and a total sample of 409 was included. See Supplemental Figure 1 for the flow of participants through the trial. Of the included respondents, a majority—350 individuals (86%)—were enrolled after learning about the study through a newspaper advertisement or recommendations from friends or family. In addition, 29 respondents (7%) discovered the study via social media, 23 (6%) learned about it at a cardiology clinic, and 7 (2%) reported learning about the study within primary care. Among the 27 cardiology clinics that were contacted by the research team, 13 clinics from across Sweden were reported as the places where respondents learned about the study. Supplemental Table 4 reports baseline characteristics depending on the recruitment channel. A majority of respondents reported that their AF was currently managed in tertiary care (n = 231, 57%) or primary care (n = 159, 39%). A small proportion (n = 19, 5%) either did not respond or indicated that they were not under active follow-up.

### Sample

The sample of 409 respondents included 251 men (61%) with a mean age of 70 ± 9.6 years. Paroxysmal AF was the most frequently reported AF type (179 respondents, 44%), followed by permanent AF (139 respondents, 34%). Persistent AF was reported by 35 respondents (9%), whereas 56 respondents (14%) were uncertain of their AF type. The average duration since AF diagnosis was 8.1 ± 8.2 years. The mean AFEQT score for the whole sample was 73.8 ± 18.97, corresponding to mild AF impairment,^23^ and the mean CAQ score was 21.8 ± 10.8. In the total sample, previous ablation for AF was reported by 58 (14%), and the mean number of medications taken was 3.0 ± 1.7. Antiarrhythmics were used by 69 (17%), beta blockers by 313 (77%), and anticoagulants by 359 (88%). For the participants with symptom preoccupation, previous ablation for AF was reported by 27 (18%), and the mean number of medications taken was 3.2 (±2). Antiarrhythmics were used by 32 (21%), beta blockers by 127 (85%), and anticoagulants by 120 (80%). Table 1 provides details on sociodemographic variables, clinical factors, and comorbidities, whereas Table 2 presents the outcomes for the dependent variables.

**Table 1:** Respondents' Characteristics

	Total Sample (N = 409)	Respondents with Symptom Preoccupation (n = 150)	Respondents without Symptom Preoccupation (n = 259)
Age -yr	70.4 ± 9.6	68.6 ± 10.7	71.4 ± 8.7
Sex			
Female	158 (39)	93 (62)	65 (25)
Male	251 (61)	57 (38)	194 (75)
AF duration, y	8.1 (8.2)	7.1 (7)	8.7 (8.8)
Employment status			
Employed	97 (24)	41 (27)	63 (24)
Retired	317 (78)	108 (72)	209 (81)
Unemployed	9 (2)	3 (2)	9 (2)
Studying	3 (1)	0 (0)	3 (1)
Sick leave	3 (1)	2 (1)	1 (0)
Highest completed education			
Prehigh school	48 (12)	20 (13)	28 (11)
High school	70 (17)	24 (16)	46 (18)
Posthigh school (not university)	63 (15)	19 (13)	44 (17)
University	213 (52)	81 (54)	132 (51)
Doctoral degree	8 (2)	3 (2)	5 (2)
Unknown	7 (2)	3 (2)	4 (2)
Type of AF			
Paroxysmal	179 (44)	87 (58)	92 (36)
Persistent	35 (9)	15 (10)	20 (8)
Chronic	139 (34)	33 (22)	106 (41)
Do not know	56 (14)	15 (10)	41 (16)
Ablation AF	58 (14)	27 (18)	31 (12)
Medical comorbidity			
Medical comorbidity any	325 (80)	186 (80)	
Sleep apnea	69 (17)	30 (20)	39 (15)
Diabetes	36 (9)	16 (11)	10 (8)
Stroke or TIA	35 (9)	16 (11)	19 (7)
Hypertension	173 (42)	72 (48)	101 (39)
Heart failure	36 (9)	20 (13)	16 (6)
Angina pectoris	14 (3)	8 (5)	6 (2)
Coronary artery disease	21 (5)	9 (6)	12 (5)
COPD	19 (4.7)	9 (6)	10 (4)
Thyroidism	28 (7)	18 (12)	10 (4)
BMI	27.1 ± 5.6	28.3 ± 7.1	26.4 ± 4.5
Obese, BMI >30 kg/m^2^	87 (21)	45 (30)	42 (16)
Functional comorbidity	39 (10)	27 (18)	12 (5)
IBS	35 (9)	25 (17)	10 (4)
Fibromyalgia	7 (2)	5 (3)	2 (1)
Mental health status			
Depression- clinically significant (PHQ-9 ≥10)	25 (6)	14 (9)	4 (2)
General anxiety- clinically significant (GAD-7 ≥8)	15 (4)	12 (8)	3 (1)
Current medication			
Number of medications (all)	3 ± 1.7	3.2 ± 2	2.9 ± 1.5
Antiarrhythmics	69 (17)	32 (21)	37 (14)
Beta-blockers	313 (77)	127 (85)	190 (73)
Anticoagulant	359 (88)	120 (80)	239 (92)
Blood pressure	126 (31)	44 (29)	82 (32)
Antidepressants	11 (3)	7 (5)	4 (2)
Sleep medication	27 (7)	13 (9)	14 (5)
Anxiolytics	5 (1)	4 (3)	1 (0)

AF = atrial fibrillation; BMI = body mass index; COPD = chronic obstructive pulmonary disease; GAD-7 = Generalized Anxiety Disorder 7-item version; IBS = irritable bowel syndrome; PHQ-9 = Patient Health Questionnaire 9-item version; TIA = transient ischemic attack.

**Table 2:** AF Disability

	Total Sample (N = 409)	Respondents With Symptom Preoccupation (N = 150)	Respondents Without Symptom Preoccupation (n = 259)
AF-specific QoL (AFEQT)	73.8 (18.7)	60.2 (19)	81.8 (13.7)
General Disability (WHODAS 2.0)	9.9 (12.3)	17.1 (14.6)	5.8 (8.3)
AF symptom severity (AFSS)	8 (6.8)	12.3 (7.4)	5.6 (4.9)
AF-specific healthcare visits (AFSS visits), last 3 mo	0.6 (1.3)	1.1 (1.7)	0.4 (0.9)

Values are mean ± SD.

AFEQT = Atrial Fibrillation Effect on Quality-of-Life; AFSS = The University of Toronto Atrial Fibrillation Severity C-scale; AFSS visits = The University of Toronto Atrial Fibrillation Severity C-scale visits items; QoL = Quality of Life; WHODAS 2.0 = World Health Organization Disability Assessment Schedule; other abbreviations as in Table 1.

### Prevalence of symptom preoccupation

Of the 409 respondents, 150 (37%) scored 26 or higher on the CAQ, meeting the threshold for clinically significant symptom preoccupation.^20^ In this group of respondents, the AFEQT mean score was 60.2 ± 19, which corresponds to moderate AF-related impairment.^26^ Among the female participants, 93 of 158 (59%) participants met the threshold for clinically significant symptom preoccupation, compared to 57 of 251 (23%) male participants.

## Results from the multiple regression analyses

Table 3 presents the results of the multiple regression analysis for the association between the independent variables and AF-specific QoL, general disability, and AF-related symptom severity. Results including all covariates are reported in Supplemental Table 1.

**Table 3:** Association of Psychological Factors and Mean Score on AF-Specific QoL (AFEQT) General Disability (WHODAS 2.0), and AF Symptom Severity (AFSS)

	AFEQT			WHODAS 2.0			AFSS		
	**β** (95% CI) Standardized	SE (Robust)	*P* Value	**β** (95% CI) Standardized	SE (Robust)	*P* Value	**β** (95% CI) Standardized	SE (Robust)	*P* Value
CAQ	−0.51 (−0.61 to −0.41)	0.05	<0.001	0.31 (0.19-0.43)	0.06	<0.001	0.39 (0.27-0.50)	0.06	<0.001
PHQ-9	−0.23 (−0.34 to −0.13)	0.06	<0.001	0.45 (0.32-0.58)	0.06	<0.001	0.28 (0.16-0.40)	0.06	<0.001
GAD-7	−0.03 (−0.14 to 0.08)	0.06	0.644	0.05 (−0.08-0.17)	0.06	0.463	0.04 (−0.07 to 0.15)	0.06	0.431
BSQ	−0.01 (−0.09 to 0.07)	0.04	0.796	−0.02 (−0.12 to 0.07)	0.05	0.663	−0.08 (−0.17 to 0.17)	0.05	0.109
ASI	−0.03 (−0.14 to 0.08)	0.06	0.623	−0,01 (−0.11 to 0.12)	0.06	0.924	0.07 (−0.05 to 0.19)	0.06	0.226
PSS4	0.01 (−0.07 to 0.09)	0.04	0.775	0.04 (−0.03 to 0.11)	0.04	0.242	0.03 (−0.05 to 0.11)	0.04	0.498
Age	−0.01 (−0.09 to 0.07)	0.04	0.802	0.08 (−0.01 to 0.16)	0.04	0.068	0.04 (−0.05 to 0.12)	0.04	0.375
AF duration	0.03 (−0.03 to 0.09)	0.03	0.327	−0.01 (−0.08 to 0.06)	0.04	0.699	−0.06 (−0.13 to 0.01)	0.04	0.081
Gender^a^	0.16 (0.01-0.32)	0.08	0.037	0.01 (−0.15 to 0.18)	0.08	0.887	−0.12 (−0.29 to 0.06)	0.09	0.190

Standardized regression coefficients. 95% CIs, *robust SEs*, and *P* values are reported from 3 separate regression analyses. AFEQT model's R^2^ = 0.609, model's *P* < 0.001. WHODAS model's R^2^ = 0.556, model's *P* < 0.001. AFSS model's R^2^ = 0.566, model's *P* < 0.001. Other sociodemographic and medical covariates were included in the models but are not reported in the table. Results including all covariates are reported in Supplemental Table 1.

ASI = Anxiety Sensitivity Index; BSQ = Bodily Sensations Questionnaire; CAQ = Cardiac Anxiety Questionnaire; PHQ-9 = Patient Health Questionnaire 9 item version; PSS4 = Perceived Stress Scale − 4-item version; other abbreviations as in Table 1 and 2.

a Unstandardized beta values for binary variable.

### AF-specific quality of life

The model for AF-specific QoL (AFEQT) showed a good overall fit, with 61% of the variance explained by the variables in the model (F [31, 377] = 21.63, *P* < 0.001). Symptom preoccupation (CAQ) showed the strongest association (β: −0.51; 95% CI: −0.61 to −0.41; *P* < 0.001). The interpretation of the β is that 1-SD increase in CAQ score was associated with a 0.51 SD decrease in AFEQT score. Depression (PHQ-9) was also associated with reduced AF-specific QoL, although the relationship was weaker (β: −0.23; 95% CI: −0.34 to −0.13; *P* < 0.001). None of the other independent variables showed statistically significant associations.

### General disability

The model explained 56% of the variance in general disability (WHODAS 2.0), with a satisfactory overall fit (F [31, 377] = 9.51; *P* < 0.001). Symptom preoccupation (β: 0.31; 95% CI: 0.19-0.43; *P* < 0.001) and depression (β: 0.45; 95% CI: 0.32-0.58; *P* < 0.001) both showed significant associations with greater general disability. No other independent variable showed a statistically significant association with general disability.

### AF symptoms

Regarding AF-related symptom severity (AFSS), the variables in the model accounted for 57% of the variance with a satisfactory overall fit of the model (F [31, 377] = 15.74; *P* < 0.001). Symptom preoccupation (β: 0.39; 95% CI: 0.27-0.50; *P* < 0.001) and depression (β: 0.28; 95% CI: 0.16-0.40; *P* < 0.001) were found to have significant associations with AF symptom severity. No statistically significant associations were observed for any other independent variable.

### AF-specific health care consumption

Table 4 presents the Poisson analysis results for associations between the independent variables and AF-specific health care consumption. Full results including all covariates are reported in Supplemental Table 2. About 12% of the variance in AF-specific health care consumption was explained by the variables in the model (Wald chi^2^ [3] = 138.66; *P* < 0.001). Each increase by one SD in symptom preoccupation was associated with an increase of AF-specific health care consumption by 67% (IRR: 1.67; 95% CI: 1.28; 2.19, *P* < 0.001). No other independent variables showed statistically significant associations with AF-specific health care consumption.

**Table 4:** Association of Psychological Factors and Incidence of AF-specific Health Care Visits During the Last 3 months (AFSS Visits)

	AF Related Healthcare Visits	95% CI for **β** Standardized	Standard Error (robust)	IRR	95% CI for IRR	*P* Value
	**β** Standardized					
CAQ	0.51	0.24-0.78	0.14	1.67	1.28-2.19	<0.001
PHQ-9	0.10	−0.15 to 0.35	0.13	1.11	0.87-1.42	0.413
GAD-7	−0.11	−0.31 to 0.35	0.10	0.93	0.73-1.10	0.294
BSQ	−0.18	−0.37 to 0.01	0.10	0.84	0.69-1.01	0.056
ASI	−0.02	−0.29 to 0.25	0.14	0.98	0.75-1.29	0.881
PSS4	0.20	−0.2 to 0.42	0.11	1.22	0.98-1.52	0.071
Age	0.03	−0.20 to 0.25	0.11	1.03	0.82-1.30	0.817
AF duration	−0.24	−0.58 to 0.10	0.17	0.78	0.56-1.10	0.160
AF episodes frequency	0.03	−0.17 to 0.23	0.10	1.03	0.85-1.26	0.753
Sex^a^	−0.11	−0.43 to 0.22	0.17	0.90	0.65-1.24	0.526

Standardized regression coefficients, robust SEs with corresponding 95% CIs, incidence rate ratios with corresponding 95% CIs and *P* values are reported from a Poisson regression analysis. model's pseudo R^2^ = 0.120, model's *P* < 0.001. Other sociodemographic and medical covariates were included in the models but are not reported in the table. Results including all covariates are reported in Supplemental Table 2.

IRR = incidence rate ratio; other abbreviations as in Table 1, 2, and 3.

a Unstandardized beta for binary variable.

### Secondary analysis of the influence of cardiac-related fear, hypervigilance/attention and avoidance on AF-specific QOL

See Supplemental Table 3. To examine the unique contribution of the different facets of symptom preoccupation on AF-related QoL (AFEQT), a linear multiple regression analysis was performed including the 3 CAQ subscales: cardiac-related fear, hypervigilance, and avoidance. All 3 subscales of CAQ showed statistically significant associations with impaired AF-related QoL (fear [β: 0.18; 95% CI: −0.27 to −0.08; *P* <0.001], hypervigilance [β: 0.24; 95% CI: −0.33 to −0.16; *P* < 0.001] and avoidance [β: 0.25; 95% CI: −0.34 to −0.16; *P* < 0.001]). Similarly to the previous analysis that included the total score of CAQ, depression (PHQ-9) (β: 0.23; 95% CI: −0.34 to −0.12; *P* < 0.001) showed a significant association with impaired AF-related QoL.

## Discussion

### Summary of primary findings

In this cross-sectional study of 409 patients with AF, we investigated the prevalence of symptom preoccupation and its relationship with AF-related QoL, general disability, self-rated AF symptom severity, and AF-specific health care consumption, compared to general psychological distress (eg, depression, general anxiety, fear of bodily sensations, anxiety sensitivity, and perceived stress). We observed clinically significant levels of symptom preoccupation (cardiac-related fear, hypervigilance, and avoidance behavior), in 37% of the total sample, with 23% of the men and 59% of the women meeting the criteria, indicating that symptom preoccupation was 2.5 times more prevalent in women. Among the psychological factors compared in this model, symptom preoccupation demonstrated the strongest associations with impaired AF-related QoL, increased AF-specific health care consumption (see Central Illustration), and AF symptom severity. Symptom preoccupation was also significantly associated with impaired general disability, although depression showed a stronger association. Moreover, each of the CAQ subscales—fear, hypervigilance, and avoidance—demonstrated independent associations with impaired AF-related QoL, supporting the hypothesis that all 3 facets of symptom preoccupation are important and independently contribute to impaired AF-related QoL.

**Central Illustration:**
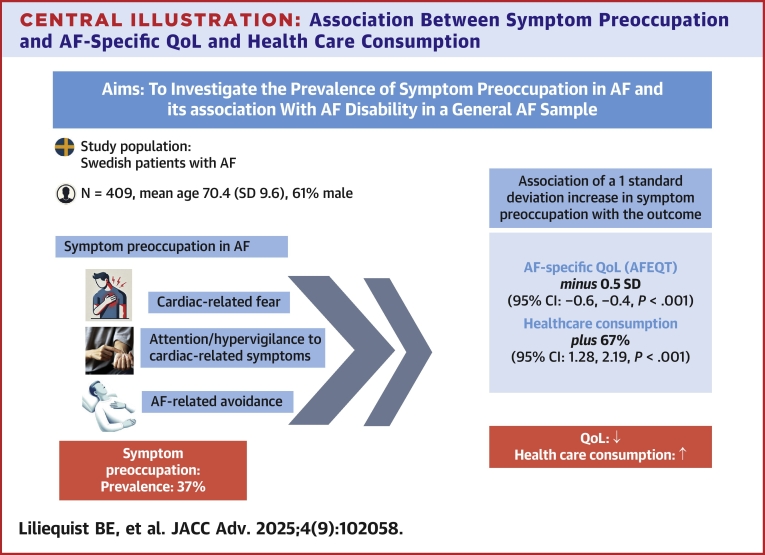
Association Between Symptom Preoccupation and AF-Specific QoL and Health Care Consumption AF = atrial fibrillation; AFEQT = atrial fibrillation effects on quality of life; QoL = quality of life.

### Comparison to other findings

The results of this study align with the previous results of Ong et al^10^ who reported symptom preoccupation as an important psychological factor linked with increased self-rated AF-symptom severity. Furthermore, the high prevalence and associations between symptom preoccupation and AF-disability in this general AF sample, extends the findings from recent studies of AF-CBT targeting symptom preoccupation in patients with paroxysmal AF,^14^^,^^16^^,^^17^ suggesting AF-CBT could be an effective adjunctive treatment for a broad range of patients with symptomatic AF. Interestingly, in line with other AF studies,^5^ we observed a significant association between female sex and impaired AF-specific QoL, even after accounting for the higher prevalence of symptom preoccupation among women. This indicates that female sex impacts QoL beyond differences in symptom preoccupation and underscores the need to consider sex differences in AF care.

In contrast to other studies in the field, we did not observe strong relationships between depression or general anxiety and AF-symptom severity,^9^^,^^29^ nor between general anxiety and impaired QoL.^30^ However, in line with Charitakis et al,^29^ depression was linked to impaired AF-specific QoL, increased AF-symptom severity, and general disability. General anxiety was not a significant independent variable for any of the outcomes.

Another difference in this sample was the low prevalence of clinically significant depression (6%) and general anxiety (15%) compared to prior studies.^31^^,^^32^ For example, Thrall et al reported rates of 38% depression and 28% anxiety in patients receiving care at cardiology clinics.^8^ Moreover, AF-related QoL and general disability was mildly impaired in our sample. These differences in the study results may be attributed to the use of a convenience sample that includes patients beyond those seen in tertiary care settings.

Despite low levels of depression and anxiety in our sample, a substantial proportion of respondents reported clinically significant symptom preoccupation. This suggests that general tendencies for anxiety, anxiety sensitivity, stress, or fear of bodily symptoms have limited relevance for AF outcomes when AF-specific symptom preoccupation is considered. This is an important finding, because it suggests that patients' ability to cope with AF is not primarily determined by general psychological factors but rather that cardiac-related fear, hypervigilance, and avoidance behavior play a more critical role in driving impairment and disability. These factors may exacerbate symptoms, reinforce fear, and restrict daily activities, thereby diminishing QoL. Consequently, psychosocial interventions for AF patients may be most effective if they target this behavioral pattern rather than general psychopathology. Furthermore, in a recent randomized controlled trial, AF-CBT led to large improvements of AF-specific QoL and reduced health care seeking.^16^ However, this was not accompanied by reductions in AF-burden or changes in heart rate variability and physical activity.^16^^,^^33^ This may further emphasize the importance of AF-specific psychological factors such as fear, hypervigilance, and avoidance.

We have proposed the term symptom preoccupation to describe this behavioral pattern that negatively impacts AF-related QoL and AF-specific health care consumption. Although the term *cardiac anxiety* has been used in other heart diseases,^20^ we argue that symptom preoccupation better captures the broader experience in AF. Our clinical experience^14^^,^^16^^,^^17^ is that not all patients with AF describe anxiety toward their symptoms and disease, rather, they describe them as frustrating or limiting of their daily functioning. Indeed, our findings demonstrate that the 3 facets of symptom preoccupation, that is, fear, hypervigilance, and avoidance behavior, each uniquely contribute to impaired AF-specific QoL, supporting the use of this broader term, which may resonate with more patients than cardiac anxiety alone.

### Strengths and limitations

The strength of the study is its use of validated AF-specific instruments to assess various dimensions of impairment and disability, alongside both general and disease-specific measures of psychological distress in a general AF sample. There are also several limitations to the present study. The use of a cross-sectional design limits our ability to infer causality and directions of our correlations. The sample recruitment based on self-referral potentially introduces a risk of selection bias that limits the generalizability of these findings to the broader AF population. Patients with AF experiencing greater symptom severity or higher levels of symptom preoccupation may have been more inclined to participate, and thus the proportion of participants showing clinically significant levels of symptom preoccupation may not generalize to the whole AF population. The financial compensation offered in this study may have incentivized participation among individuals with lower socioeconomic status. However, as the sample predominantly consisted of highly educated respondents, we consider the risk of significant bias to be minimal. The inclusion of patient-reported outcome measures on, for example, QoL and symptom severity is strongly advocated in arrhythmia research and clinical care.^34^ Nevertheless, a potential limitation is the reliance on self-reported information regarding medical history and AF diagnosis. Although self-reports of chronic diseases are generally considered acceptable and reliable in many studies,^18^ this approach may still introduce a degree of uncertainty—particularly regarding the type of AF diagnosed. This is compounded by the well-documented tendency of patients to both underestimate and overestimate their symptoms,^35^ and to fluctuate between different AF classifications over time. Most respondents completed the survey after March 11, 2020, when the World Health Organization declared COVID-19 a pandemic. This may have increased anxiety and impaired QoL in our sample of respondents with AF. However, no pandemic-related restrictions with potential QoL implications were in place in Sweden at that time. Moreover, levels of anxiety and depression in this sample were lower than those reported in other studies of patients with AF, suggesting that the overall impact of the pandemic on psychological outcomes in this cohort was limited.

## Conclusions

Our results show that symptom preoccupation is common among patients with AF, especially among women, and independently associated with AF-specific QoL, general disability, AF symptom severity, and AF-specific health care consumption. These results highlight the importance of targeting symptom preoccupation in patients with AF and may lead to a better understanding of patients with AF and to the development of additive treatment strategies to target symptom preoccupation in the management of AF. Thus, interventions targeting cardiac-specific behaviors related to fear, hypervigilance, and avoidance in AF should be made accessible and integrated in a multidisciplinary approach to AF care.

Perspectives **COMPETENCY IN MEDICAL KNOWLEDGE:** Symptomatic AF is associated with distressing symptoms and impaired QoL. Current rhythm and rate control treatments may provide limited relief in many patients.

**COMPETENCY IN PATIENT CARE:** The results of this study show that symptom preoccupation, that is, fear of cardiac-related symptoms and avoidance behavior is common and associated with AF-specific QoL and health care use. These insights should ideally be integrated into multidisciplinary concepts of AF care, including standardized AF education and screening for symptom preoccupation in patients with AF.

**TRANSLATIONAL OUTLOOK:** The results add further support for integrating treatments targeting symptom preoccupation, such as cognitive behavioral therapy, into AF care to reduce AF-related disability.

## Funding support and author disclosures

This study was supported by research and development grants from Karolinska University Hospital, Stockholm Sweden. The funding body was not involved in the design of the study, data analysis, or interpretation of the results. Dr Braunschweig declares personal fees for trial committee participation and lectures by Medtronic, Biotronik, Biosense Webster, Impulse Dynamics, Novartis, Orion, Boehringer, and Pfizer. Dr Ljótsson has co-authored a Swedish self-help book for health anxiety and has a publishing agreement with Cambridge University Press for a self-help book for irritable bowel syndrome. All other authors have reported that they have no relationships relevant to the contents of this paper to disclose.
