# Supplementary material for: Symptom Preoccupation in Atrial Fibrillation and its Association With Quality of Life: A Cross-Sectional Study
Source: JACC Adv. 2025 Aug 8;4(9):102058. doi: 10.1016/j.jacadv.2025.102058 (PMC12356299; doi:10.1016/j.jacadv.2025.102058)
Supplement: Supplementary data [file mmc1.docx]

**Supplemental Appendix**

Symptom Preoccupation in Atrial Fibrillation and Its Association With
Impaired Quality of Life: A Cross-Sectional Study

This supplemental material has been provided by the authors to give readers additional information about their work.

Contents

[**Self-report of diagnosis and classification of atrial fibrillation** 2](#_Toc198803186)

[**Supplemental Figure 1. Flow of the participants through the study (CONSORT diagram)** 3](#_Toc198803187)

[**Supplemental Table 1. Association of psychological factors and mean score on AF-specific QoL (AFEQT) General disability (WHODAS 2.0), and AF symptom severity (AFSS)** 4](#_Toc198803188)

[**Supplemental Table 2. Association of psychological factors and incidence of AF-specific healthcare visits during the last 3 months (AFSS visits)** 7](#_Toc198803189)

[**Supplemental Table 3. Association between the three CAQ subscales: cardiac-related fear, hypervigilance and avoidance and AFEQT** 9](#_Toc198803190)

[**Supplemental Table 4. AF- related Quality of Life, symptom preoccupation, and mental health status based on recruitment sources** 10](#_Toc198803191)

## **Self-report of diagnosis and classification of atrial fibrillation**

The alternatives read (1) “I have been diagnosed with atrial fibrillation that comes and goes, meaning I do not have atrial fibrillation all the time (intermittent; paroxysmal atrial fibrillation), (2)” I have been diagnosed with atrial fibrillation that does not resolve on its own, lasts more than 7 days, and requires interventions such as electrical cardioversion to return to normal heart rhythm (persistent atrial fibrillation), (3) I have been diagnosed with atrial fibrillation that persists all the time (chronic atrial fibrillation), (4) I have an atrial fibrillation diagnosis, but I am not sure which type it is”

## **Supplemental Figure 1. Flow of the participants through the study (CONSORT diagram)**

**Excluded**

**(n=34)**

Did not complete survey (n=32)

Declined participation (n=2)

**Registered online**

**(n=443)**

**Included**

**(n=409)**

## **Supplemental Table 1. Association of psychological factors and mean score on AF-specific QoL (AFEQT) General disability (WHODAS 2.0), and AF symptom severity (AFSS)**

| Dependent variables | | AFEQT |  |  | WHODAS 2.0 |  |  | AFSS |  |  |
| --- | --- | --- | --- | --- | --- | --- | --- | --- | --- | --- |
| Independent variables | | β (95% CI)  Standardized | Standard error (robust) | *P* Value | β (95% CI)  Standardized | Standard error (robust) | *P* value | β (95% CI)  Standardized | Standard error (robust) | *P* value |
| CAQ | | -0.51 **(-**0.61 to -0.41) | 0.05 | < .001 | 0.31 (0.19 to 0.43) | 0.06 | < .001 | 0.39 (0.27 to 0.50) | 0.06 | < .001 |
| PHQ-9 | | -0.23 (-0.34 to -0.13) | 0.06 | < .001 | 0.45 ( 0.32 to 0.58) | 0.06 | < .001 | 0.28 (0.16 to 0.40) | 0.06 | < .001 |
| GAD-7 | | -0.03 (-0.14 to 0.08) | 0.06 | .644 | 0.05 (-0.08 to 0.17) | 0.06 | .463 | 0.04 (-0.07 to 0.15) | 0.06 | .431 |
| BSQ | | -0.01 (-0.09 to 0.07) | 0.04 | .796 | -0.02 (-0.12 to 0.07) | 0.05 | . 663 | -0.08 (-0.17 to 0.17) | 0.05 | .109 |
| ASI | | -0.03 (-0.14 to 0.08) | 0.06 | .623 | -0,01 (-0.11 to 0.12) | 0.06 | . 924 | 0.07 (-0.05 to 0.19) | 0.06 | .226 |
| PSS4 | | 0.01 (-0.07 to 0.09) | 0.04 | .775 | 0.04 (-0.03 to 0.11) | 0.04 | .242 | 0.03 (-0.05 to 0.11) | 0.04 | .498 |
| Age | | -0.01 (-0.09 to 0.07) | 0.04 | .802 | 0.08 (-0.01 to 0.16) | 0.04 | .068 | 0.04 (-0.05 to 0.12) | 0.04 | .375 |
| AF duration | | 0.03 (-0.03 to 0.09) | 0.03 | .327 | -0.01 (-0.08 to 0.06) | 0.04 | .699 | -0.06 (-0.13 to 0.01) | 0.04 | 0.081 |
| AF episodes frequency | | -0.14 (-0.22 to -0.06) | 0.04 | >.001 | 0.08 (-0.002 to 0.16) | 0.04 | .056 | 0.18 (0.11 to 0.26) | 0.04 | < .001 |
| Gender^a^ | | 0.16 (0.01 to 0.32) | 0.08 | .037 | 0.01 (-0.15 to 0.18) | 0.08 | .887 | -0.12 (-0.29 to 0.06) | 0.09 | .190 |
| Education^a^ | |  |  |  |  |  |  |  |  |  |
|  | High school^a^ | reference | - | - | reference | - | - | reference | - | - |
|  | Less than high school^a^ | -0.04 (-0.26 to 0.19) | 0.11 | .730 | -0.08 (-0.35 to 0.19) | 0.14 | .567 | -0.07 (-0.34 to 0.21) | 0.14 | .627 |
|  | Post high school/not university^a^ | 0.06 (-0.14 to 0.26) | 0.10 | .569 | -0.10 (-0.35 to 0.15) | 0.13 | .430 | -0.13 (-0.35 to 0.10) | 0.11 | .266 |
|  | University^a^ | 0.08 (-0.08, 0.24) | 0.08 | . 344 | -0.09 (-0.28 to 0.11) | 0.10 | .394 | -0.14 (-0.33 to 0.04) | 0.10 | .131 |
|  | Unknown^a^ | -0.55 (-1.1 to 0.02) | 0.29 | . 057 | 0.37 (-0.23 to 0.96) | 0.30 | . 225 | 0.48 (-0.14 to 1.10) | 0.32 | .127 |
| AF type^a^ | |  |  |  |  |  |  |  |  |  |
|  | Paroxysmal^a^ | reference | - | - | reference | - | - | reference | - | - |
|  | Persistent^a^ | -0.37 (-0.66 to -0.08) | 0.15 | .011 | 0.30 (0.04 to 0.56) | 0.13 | .026 | 0.20 (-0.09 to 0.49) | 0.15 | .179 |
|  | Permanent^a^ | -0.15 (-0.32 to 0.03) | 0.09 | . 097 | 0.21 (0.01 to 0.43) | 0.11 | .038 | 0.04 (-0.14 to 0.22) | 0.09 | .674 |
|  | Unknown^a^ | 0.64 (-0.27 to 0.14) | 0.10 | . 529 | 0.25 (0.001 to 0.51) | 0.13 | .050 | 0.08 (-0.14 to 0.29) | 0.11 | .488 |
| Heart failure^a^ | | -0.26 (-0.49 to 0.02) | 0.13 | .068 | 0.31 (-0.04 to 0.66) | 0.18 | .085 | 0.46 (0.17 to 0.76) | 0.15 | .002 |
| CAD^a^ | | -0.23 (-0.53 to 0.06) | 0.15 | .121 | -0.02 (-0.42 to 0.37) | 0.20 | .903 | 0.15 (-0.19 to 0.49) | 0.17 | .390 |
| Hypertension^a^ | | -0.08 (-0.23 to 0.07) | 0.07 | .279 | -0.14 (-0.30 to 0.02) | 0.08 | .088 | -0.12 (-0.28 to 0.04) | 0.08 | .137 |
| Sleep apnea^a^ | | -0.06 (-0.26 to 0.09) | 0.10 | .315 | 0.06 (-0.28 to 0.17) | 0.11 | .613 | -0.06 (-0.25 to 0.14) | 0.10 | .564 |
| Stroke^a^ | | -0.07 (-0.32 to 0.13) | 0.13 | .520 | 0.07 (-0.16 to 0.30) | 0.12 | .576 | 0.03 (-0.21 to 0.27) | 0.12 | .818 |
| Diabetes mellitus^a^ | | 0.001 (-0.20 to 0.20) | 0.10 | .996 | 0.13 (-0.17 to 0.43) | 0.15 | .399 | -0.05 (-0.27 to 0.18) | 0.11 | .679 |
| COPD | | -0.12 (-0.39 to 0.15) | 0.14 | .389 | 0.20 ( 0.23 to 0.63)) | 0.22 | .352 | 0.08 (-0.26 to 0.42) | 0.18 | .648 |
| Angina pectoris^a^ | | 0.07 (-0.38 to 0.52) | 0.23 | .755 | 0.51 (-0.17 to 1.18) | 0.34 | .139 | 0.59 (0.15 to 1.02) | 0.22 | .08 |
| BMI | | 0.04 (-0.12 to 0.05) | 0.04 | .440 | 0.08 ( -0.001 to 0.17) | 0.04 | .054 | 0.04 ( -0.04 to 0.12) | 0.04 | .355 |
| Thyroidism^a^ | | 0.33 (0.01 to 0.65) | 0.16 | .041 | -0.05 (-0.36 to 0.26) | 0.16 | .733 | -0.22 (-0.58 to 0.14) | 0.18 | .234 |
| Number of medications | | -0.01 (-0.15 to 0.13) | 0.07 | .869 | -0.001 (-0.14 to 0.14) | 0.07 | .994 | -0.04 (-0.18 to 0.10) | 0.07 | .544 |
| Any other medical issue^a^ | | -0.02 (-0.15 to 0.11) | 0.07 | .789 | 0.11 (0.03 to 0.24) | 0.07 | .134 | 0.11 (-0.04 to 0.26) | 0.08 | .146 |
| Functional disease^a^ | | -0.15 (-0.45 to 0.15) | 0.15 | .315 | 0.24 (-0.10 to 0.58) | 0.17 | .161 | 0.02 (-0.27 to 0.31) | 0.15 | .880 |
| Sleep disturbance | | 0.01 (-0.14 to 0.16) | 0.08 | .867 | -0.13 (-0.30 to 0.04) | 0.09 | .143 | 0.11 (-0.05 to 0.27) | 0.08 | .168 |

^a^ Unstandardized beta values for binary variable. Note: Regression coefficients. 95% confidence intervals, *t*-values and *P*-values are reported from three separate regression analysis using robust standard errors. AFEQT Model´s R^2^ =0.609 , Model´s *P*-value <.001, WHODAS Model´s R^2 =^ 0.556, Model´s *P*-value <.001. AFSS Model´s R^2^ = 0.566, Model´s *P*- value <.001. AF: Atrial Fibrillation, QoL: Quality of Life, AFEQT: Atrial Fibrillation Effect on Quality-of-Life, WHODAS 2.0: World Health Organization Disability Assessment Schedule, AFSS: The University of Toronto Atrial Fibrillation Severity C-scale, CAQ: Cardiac Anxiety Questionnaire, PHQ-9: Patient Health Questionnaire 9 item version, GAD-7: Generalized Anxiety Disorder 7 item version, BSQ: Bodily Sensations Questionnaire, ASI: Anxiety Sensitivity Index, PSS4: Perceived Stress Scale - 4 item version, CAD: Coronary Artery Disease, COPD: Chronic Obstructive Pulmonary Disease, BMI: Body Mass Index.

## **Supplemental Table 2. Association of psychological factors and incidence of AF-specific healthcare visits during the last 3 months (AFSS visits)**

| Independent variables | | AF related healthcare visits |  |  |  |  |  |
| --- | --- | --- | --- | --- | --- | --- | --- |
|  | | β  Standardized | 95% CI for β  Standardized | Standard error (robust) | IRR | 95% CI for IRR | *P* value |
| CAQ | | 0.51 | 0.24 to 0.78 | 0.14 | 1.67 | 1.28 to 2.19 | < .001 |
| PHQ-9 | | 0.10 | -0.15 to 0.35 | 0.13 | 1.11 | 0.87 to 1.42 | .413 |
| GAD-7 | | -0.11 | -0.31 to 0.35 | 0.10 | 0.93 | 0.73 to 1.10 | .294 |
| BSQ | | -0.18 | -0.37 to 0.01 | 0.10 | 0.84 | 0.69 to 1.01 | .056 |
| ASI | | -0.02 | -0.29 to 0.25 | 0.14 | 0.98 | 0.75 to1.29 | .881 |
| PSS4 | | 0.20 | -0.2 to 0.42 | 0.11 | 1.22 | 0.98 to 1.52 | .071 |
| Age | | 0.03 | -0.20 to 0.25 | 0.11 | 1.03 | 0.82 to 1.30 | .817 |
| AF duration | | -0.24 | -0.58 to 0.10 | 0.17 | 0.78 | 0.56 to1.10 | .160 |
| AF episodes frequency | | 0.03 | -0.17 to 0.23 | 0.10 | 1.03 | 0.85 to 1.26 | .753 |
| Gender^a^ | | -0.11 | -0.43 to 0.22 | 0.17 | 0.90 | 0.65 to 1.24 | .526 |
| Education^a^ | |  |  |  |  |  |  |
|  | High school^a^ | Reference |  | - | - | - | - |
|  | Less than high school^a^ | -0.19 | -0.96 to 0.59 | 0.40 | 0.83 | 0.38 to 1.80 | .637 |
|  | Post high school/not university^a^ | 0.22 | -0.43 to 0.86 | 0.33 | 1.24 | 0.65 to 2.37 | .512 |
|  | University^a^ | 0.05 | -0.52 to 0.61 | 0.29 | 1.05 | 0.60 to 1.85 | .870 |
|  | Unknown^a^ | 0.75 | -0.02 to 1.53 | 0.40 | 2.12 | 0.98 to 5.60 | .057 |
| AF type^a^ | |  |  |  |  |  |  |
|  | Paroxysmal^a^ | Reference |  |  | - | - | - |
|  | Persistent^a^ | 0.89 | 0.45 to 1.34 | 0.23 | 2.44 | 1.56 to 3.80 | < .001 |
|  | Permanent^a^ | -0.26 | -0.77 to 0.25 | 0.26 | 0.77 | 0.47 to1.29 | .321 |
|  | Unknown^a^ | 0.16 | -0.45 to 0.76 | 0.31 | 1.17 | 0.64 to 2.15 | .611 |
| Heart failure^a^ | | 0.15 | 0.37 to 0.67 | 0.27 | 1.16 | 0.69 to 1.95 | .576 |
| CAD^a^ | | -0.39 | -1.16 to 0.38 | 0.39 | 0.68 | 0.32 to 1.46 | .318 |
| Hypertension^a^ | | 0.19 | -0.18 to 0.56 | 0.19 | 1.21 | 0.83 to 1.75 | .322 |
| Sleep apnea^a^ | | -0.08 | -0.52 to 0.36 | 0.22 | 0.93 | 0.60 to 1.44 | .733 |
| Stroke^a^ | | -0.23 | -0.90 to 0.43 | 0.34 | 0.79 | 0.41 to 1.55 | .495 |
| Diabetes mellitus^a^ | | -0.93 | -1.57 to -0.29 | 0.33 | 0.40 | 0.21 to 0.75 | .004 |
| COPD | | 0.33 | -0.37 to 1.02 | 0.36 | 1.38 | 0.69 to 2.78 | .360 |
| Angina pectoris^a^ | | 0.21 | 0.55 to 0.98 | 0.39 | 1.24 | 0.58 to 2.66 | .588 |
| BMI | | 0.003 | -0.17 to 0.18 | 0.89 | 1.00 | 0.84 to 1.19 | .969 |
| Thyroidism^a^ | | -0.33 | -1.05 to 0.40 | 0.37 | 0.72 | 0.35 to 1.48 | .363 |
| Number of medications | | 0.29 | -0.15 to 0.60 | 0.16 | 1.34 | 0.99 to 1.83 | .062 |
| Any other medical issue^a^ | | -0.14 | -0.52 to 0.25 | 0.20 | 0.87 | 0.60 to 1.30 | .487 |
| Functional disease^a^ | | 0.59 | -0.05 to 1.22 | 0.33 | 1.80 | 0.95 to 3.40 | .072 |
| Sleep disturbance | | -0.052 | -0.42 to 0.32 | 0.19 | 0.95 | 0.66 to 1.38 | .001 |

^a^ Unstandardized beta values for binary variables. **Note:** Regression coefficients, incidence rate rations and corresponding 95% confidence intervals and *P* values are reported from a Poisson regression analysis using robust standard errors. Model´s pseudo R^2^ = 0.120, Model´s *P* value: < .001**. Abbreviations**: AF: Atrial Fibrillation, AFSS visits: The University of Toronto Atrial Fibrillation Severity C-scale visits items, IRR: Incidence Rate Ratio, CAQ: Cardiac Anxiety Questionnaire, PHQ-9: Patient Health Questionnaire 9 item version, GAD-7: Generalized Anxiety Disorder 7 item version, BSQ: Bodily Sensations Questionnaire, ASI: Anxiety Sensitivity Index, PSS4: Perceived Stress Scale - 4 item version, CAD: Coronary Artery Disease, COPD: Chronic Obstructive Pulmonary Disease, BMI: Body Mass Index.

## **Supplemental Table 3. Association between the three CAQ subscales: cardiac-related fear, hypervigilance and avoidance and AFEQT**

|  | | AFEQT |  |  |
| --- | --- | --- | --- | --- |
| Independent variables | | β (95% CI)  Standardized | Standard error (robust) | *P* value |
| CAQ fear | | -0.18 (-0.27 to -0.08) | 0.05 | < .001 |
| CAQ hypervigilance | | -0.24 (-0.33 to -0.16) | 0.05 | < .001 |
| CAQ avoidance | | -0.25 (-0.34 to -0.16) | 0.05 | < .001 |
| PHQ-9 | | -0.23 (-0.34 to -0.12) | 0.06 | < .001 |
| GAD-7 | | -0.03 (-0.14 to 0.08) | 0.06 | .583 |
| BSQ | | -0.02 (-0.11 to 0.06) | 0.04 | .574 |
| ASI | | -0.03 (-0.14 to 0.08) | 0.06 | .540 |
| PSS4 | | 0.01 (-0.07 to 0.09) | 0.04 | .840 |
| Age | | -0.01 (-0.09 to 0.07) | 0.04 | .826 |
| Duration | | 0.04 (-0.03 to 0.10) | 0.03 | .243 |
| AF episodes frequency | | -0.14 (-0.22 to -0.06) | 0.04 | < .001 |
| Gender^a^ | | 0.15 (-0.0003 to 0.30) | 0.08 | .050 |
| Education^a^ | |  |  |  |
|  | High school^a^ | reference | - | - |
|  | Less than high school^a^ | -0.06 (-0.28 to 0.16) | 0.11 | .616 |
|  | Post high school/not university^a^ | 0.06 (-0.15 to 0.26) | 0.10 | .592 |
|  | University^a^ | 0.07 (-0.09 to 0.23) | 0.08 | .369 |
|  | Unknown^a^ | -0.59 (-1.20 to 0.02) | 0.30 | .057 |
| AF type^a^ | |  |  |  |
|  | Paroxysmal^a^ | reference | - | - |
|  | Persistent^a^ | -0.38 (-0.67 to -0.09) | 0.15 | .010 |
|  | Permanent^a^ | -0.16 (-0.34 to 0.02) | 0.09 | .082 |
|  | Unknown^a^ | -0.08 (-0.28 to 0.13) | 0.10 | .466 |
| Heart failure^a^ | | -0.24 (-0.50 to 0.01) | 0.13 | .060 |
| CAD^a^ | | -0.21 (-0.50 to 0.08) | 0.15 | .152 |
| Hypertension^a^ | | -0.09 (-0.23 to 0.06) | 0.07 | .255 |
| Sleep apnea^a^ | | -0.06 (-0.26 to 0.13) | 0.10 | .522 |
| Stroke^a^ | | -0.08 (-0.32 to 0.17) | 0.12 | .531 |
| Diabetes mellitus^a^ | | -0.01 (-0.21 to 0.19) | 0.10 | .928 |
| COPD | | -0.12 (-0.39 to 0.15) | 0.15 | .389 |
| Angina pectoris^a^ | | 0.07 (-0.39 to 0.52) | 0.23 | .775 |
| BMI | | -0.04 (-0.13 to 0.05) | 0.05 | .440 |
| Thyroidism^a^ | | 0.31 (0.003 to 0.62) | 0.16 | .052 |
| Number of medications | | -0.002 (-0.14 to 0.13) | 0.07 | .979 |
| Any other medical issue^a^ | | -0.01 (-0.14 to 0.12) | 0.07 | .865 |
| Functional disease^a^ | | -0.15 (-0.44 to 0.15) | 0.15 | .340 |
| Sleep disturbance | | 0.04 (-0.12 to 0.19) | 0.08 | .640 |

^a^ Unstandardized beta values for binary variables. **Note:** Standardized regression coefficients, 95% confidence intervals, robust standard errors and *P* values are reported from a regression analysis. AFEQT Model´s R^2^ =0.614 , Model´s *P* value <.001**. Abbreviations:** CAQ: Cardiac Anxiety Questionnaire, AFEQT: Atrial Fibrillation Effects on Quality-of-Life, PHQ-9: Patient Health Questionnaire 9 item version, GAD-7: Generalized Anxiety Disorder 7 item version, BSQ: Bodily Sensations Questionnaire, ASI: Anxiety Sensitivity Index, PSS4: Perceived Stress Scale - 4 item version, AF: Atrial Fibrillation, CAD: Coronary Artery Disease, COPD: Chronic Obstructive Pulmonary Disease, BMI: Body Mass Index.

## **Supplemental Table 4. AF- related Quality of Life, symptom preoccupation, and mental health status based on recruitment sources**

|  | **Total sample**  **(n = 409)** | **Cardiology clinic**  **(n = 23)** | **Primary care**  **(n = 7)** | **Advertisement in local newspaper**  (**n = 350)** | **Social media**  **(n = 29)** |
| --- | --- | --- | --- | --- | --- |
| WHODAS 2.0 | 10 ±12.3 | 9.4 ± 10.2 | 10.2 ± 12.8 | 10 ± 12.3 | 10.3 ± 13.4 |
| AFEQT | 73.9 ±19 | 72 ±18.5 | 60.8 ± 21.3 | 74.4 ± 8.5 | 71.4 ± 23.6 |
| CAQ | 21.9 ±10.8 | 22.1± 13.3 | 26.1 ± 10.5 | 21.5 ± 10.6 | 25 ± 11 |
| Depression- clinically significant  ( PHQ-9 ≥ 10) | 25 (6) | 0 | 1 (14) | 22 (6) | 2 (7) |
| General anxiety- clinically significant (GAD-7 ≥ 8) | 15 (4) | 0 | 1 (14) | 13 (4) | 1 (4) |

**Abbreviations:** WHODAS 2.0: World Health Organization Disability Assessment Schedule 2.0, AFEQT: Atrial Fibrillation Effect on Quality-of-Life, CAQ: Cardiac Anxiety Questionnaire, PHQ-9: Patient Health Questionnaire 9 item version, GAD-7: Generalized Anxiety Disorder 7 item version.
